# Case Report: Whole-genome sequencing of urothelial carcinoma in an adult patient with CLOVES syndrome reveals a lack of *PIK3CA* mutation and a genomic landscape consistent with urothelial carcinoma

**DOI:** 10.3389/fonc.2026.1704090

**Published:** 2026-02-20

**Authors:** Lauren McAuley, Orla Fitzpatrick, Nicola Cosgrove, Jad Yacoub, Liam Grogan, Bryan T. Hennessy, Simon J. Furney, Sinead Toomey

**Affiliations:** 1 Genomic Oncology Research Group, Department of Physiology and Medical Physics, Royal College of Surgeons in Ireland, Dublin, Ireland; 2 Department of Medical Oncology, Beaumont Hospital, Dublin, Ireland; 3 Cancer Clinical Trials and Research Unit, Beaumont Hospital, Dublin, Ireland; 4 Medical Oncology Group, Department of Molecular Medicine, Royal College of Surgeons in Ireland, Dublin, Ireland

**Keywords:** cancer, case report, CLOVES syndrome, *PIK3CA*, *PIK3CA*-related overgrowth spectrum, urothelial carcinoma

## Abstract

Congenital lipomatous overgrowth, vascular epidermal nevi, and skeletal abnormalities (CLOVES) syndrome is a rare genetic disorder caused by somatic activating mutations in the *PIK3CA* gene that arise during embryonic development. Mutations in the PI3K–AKT–mTOR pathway have been linked to various benign overgrowth disorders, including syndromes within the *PIK3CA*-related overgrowth spectrum. Somatic *PIK3CA* mutations also occur frequently across many cancer types; however, evidence linking CLOVES syndrome to increased cancer risk is not conclusive. Here, we describe a whole-genome sequencing (WGS) study of a primary pT3 high-grade urothelial carcinoma in a 62-year-old male patient diagnosed with CLOVES syndrome. A left laparoscopic nephroureterectomy was completed. Tumour tissue and a matched blood sample were collected for whole-genome sequencing, and somatic variant detection was performed. The somatic alterations were consistent with previous reports of urothelial carcinoma, including homozygous deletions of *CDKN2A* and *CDKN2B*. In this case, we could not detect somatic *PIK3CA* alterations in this patient’s urothelial carcinoma and suggest that it is unrelated to CLOVES syndrome.

## Background

Congenital lipomatous overgrowth, vascular epidermal nevi, and skeletal abnormalities (CLOVES) syndrome is a rare genetic disorder that is characterised by lipomatous overgrowth, vascular malformations, and skeletal abnormalities. CLOVES syndrome is caused by somatic activating mutations in the *PIK3CA* gene that arise during embryonic development. Thus, *PIK3CA* mutations in CLOVES syndrome are mosaic in nature.

*PIK3CA* mutations are well-known drivers of oncogenesis. *PIK3CA* encodes p100α, the catalytic subunit of the phosphoinositide 3-kinase (PI3K) enzyme. PI3K activates signalling through the AKT–mTOR axis to promote cell growth, metabolism, and cellular survival. Hyperactivating mutations in *PIK3CA* occur in approximately 11% of all cancers, with several hotspot mutations in exons 9 (E542K and E545K) and 20 (H1047R and H1047L) frequently observed (1). The mutational landscape of *PIK3CA* in CLOVES syndrome shares some similarities with that of cancer. Both hyperactivating hotspot *PIK3CA* mutations and non-hotspot mutations with lesser functional consequences occur in CLOVES syndrome (2, 3). In CLOVES syndrome, mutations in *PIK3CA* tend to be present at lower frequencies than in cancer due to the mosaic nature of the disorder. The genotype–phenotype relationship between hotspot/non-hotspot *PIK3CA* mutations and the clinical severity of CLOVES syndrome remains unclear, as the variant allele frequency (VAF) of *PIK3CA* mutations in CLOVES syndrome does not correlate with clinical severity, although this may be attributable to difficulties related to clinical sampling (4). Despite the link between *PIK3CA* and oncogenesis, there is limited evidence to suggest a higher risk of *PIK3CA-*driven cancers in patients with CLOVES syndrome (3, 5–7). Due to the rarity of CLOVES syndrome, further investigation is required to conclusively determine the cancer risk in this patient population. Here, we add to the literature a whole-genome sequencing (WGS) study of a primary urothelial carcinoma in a 62-year-old patient diagnosed with CLOVES syndrome.

## Case presentation

A 62-year-old Irish male patient with a clinical diagnosis of CLOVES syndrome initially presented in November 2017 with kyphosis, pectus excavatum, and unilateral pain, with numbness and atrophy of paraspinal muscles. In 2020, genetic testing was performed, but no causative mutation was identified. A molecular diagnosis of CLOVES syndrome could not be made. A repeat biopsy was scheduled with a clinical geneticist, but the patient did not attend. Electromyography and nerve conduction studies confirmed radiculopathy, and a subsequent MRI revealed lipomatous infiltrate adjacent to the erector spinae, spinal canal stenosis, and severe atrophy of spinal discs. This was associated with significant lower limb weakness, resulting in reduced mobility and severe pain requiring specialised pain team reviews. Large capillary vascular malformations were visible on clinical examination on his right flank. The patient had previously undergone lipoma excision multiple times within a different health system.

**Figure 1:**
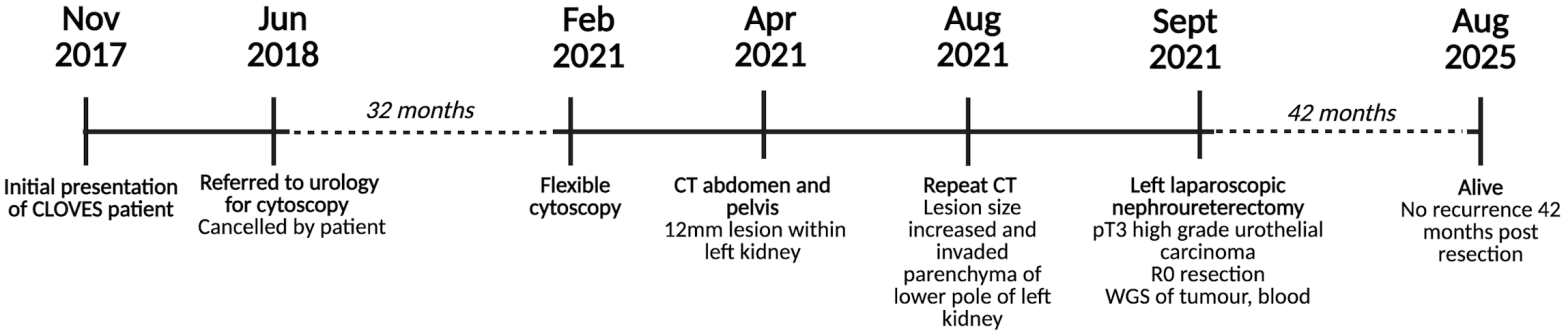
Timeline of this patient’s case.

In June 2018, the patient was referred to urology due to difficulty voiding and intermittent haematuria, persistent for 2 years. A cystoscopy was planned but cancelled by the patient. In February 2021, a flexible cystoscopy was performed after the patient presented with worsening lower urinary tract symptoms. Two months later (April 2021), CT imaging of the abdomen and pelvis identified a 12-mm lesion within the left kidney, which was radiologically concerning for urothelial carcinoma. On a repeat CT performed in August 2021, the lesion had increased in size and was now invading the parenchyma of the lower pole of the left kidney. A flexible ureteroscopy and biopsy of the lesion identified a high-grade urothelial carcinoma. In September 2021, a left laparoscopic nephroureterectomy was performed. The tumour was diagnosed as pT3 high-grade urothelial carcinoma within the renal pelvis with negative margins (R0 resection). Tumour and matched blood were collected for whole-genome sequencing. The patient elected to forgo adjuvant systemic therapy due to the risk of peripheral neuropathy associated with platinum-based chemotherapy, given his background of significant neurological deficits related to his spinal condition. Since his resection of this high-grade urothelial cancer, this patient continues with radiological, clinical, and cystoscopy follow-ups at regular intervals. He is alive and without evidence of disease recurrence 42 months post-resection. The timeline of this case is shown in **Figure 1**.

## Results

Single-region bulk whole-genome sequencing was performed for the resected tumour and matched blood with average coverages of 33× and 34×, respectively. A panel of 49 genes in which germline variants are known to increase urothelial carcinoma (UC) risk were assessed for germline variants in both the blood and tumour (8). Additionally, *PIK3CA* was also investigated for germline variants. No known pathogenic germline single-nucleotide variants (SNVs) or insertions and deletions (indels) were detected in any of these 50 genes or the associated promoters and enhancers. Additionally, no germline structural variants (SVs) or copy number variants (CNVs) affecting the 50 genes were detected. With no genetic predispositions to UC detected in the gene panel, tumour-normal analyses were performed to identify somatic SNVs, indels, multinucleotide variants (MNVs), SVs, and copy number alterations (CNAs). The details of WGS and germline/somatic variant analyses are available in the **Supplementary Material**.

### No hyperactivating somatic mutations affecting *PIK3CA* were detected

Using Mutect2 and Strelka, a total of 12,691 somatic small mutations were detected genome-wide (7,760 SNVs, 4,136 indels, and 795 MNVs). Of these, just one MNV (chr3:179207972:CC: GT) affected the *PIK3CA* gene. This double substitution is intronic and was not predicted to affect splicing or protein function using SpliceAI. No other somatic small variants affecting the *PIK3CA* gene, its promoter, or associated enhancers were detected using Mutect or Strelka. Force-calling of hotspot positions at E452, E545, H1047, and N345 of *PIK3CA* was performed using Mutect2 in tumour-only mode on both the tumour and blood samples separately in order to prevent the exclusion of any somatic mosaic variants shared between these samples. No hotspot *PIK3CA* mutations were detected in the tumour or in the blood samples. Visual inspection confirmed that no activating mutations were present at hotspot positions in *PIK3CA* in the tumour or blood (**Supplementary Figure 1**).

**Figure 2:**
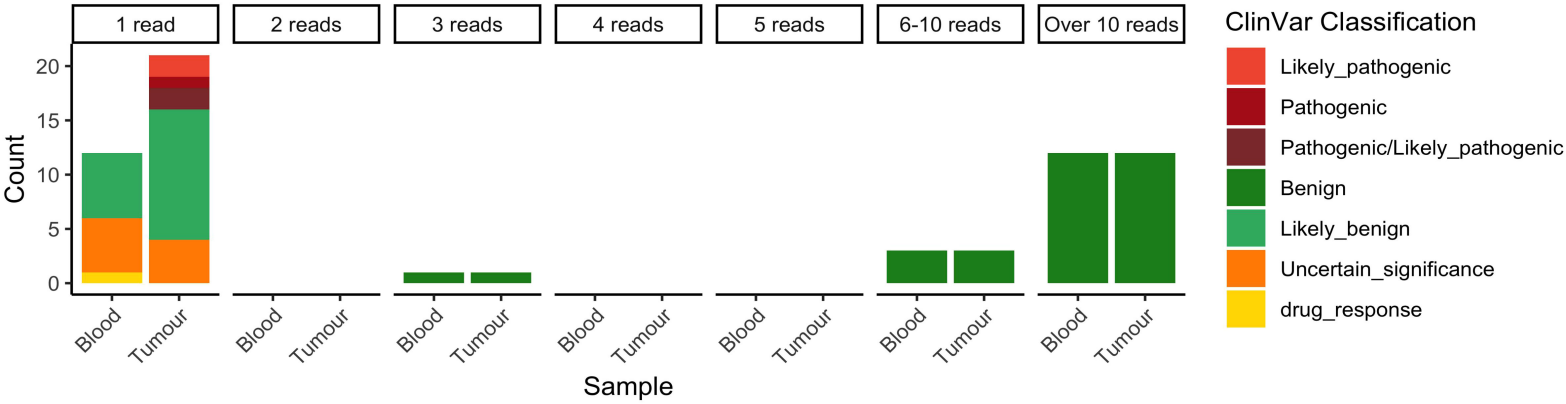
Alternate alleles reported by VarScan in blood and tumour samples with ClinVar classifications available.

To ensure that algorithmic limitations did not exclude any potential low-frequency small somatic variants, counts of nucleotides across the *PIK3CA* gene were analysed. Across the 90,754 nucleotides in the *PIK3CA* gene, 3,651 and 2,789 alternate alleles were supported by at least one read in the blood and the tumour samples, respectively. The majority of the alternate alleles were not classified in ClinVar; 19/2,789 and 28/3,651 alternate alleles detected in the blood and tumour, respectively, had available ClinVar classifications. No pathogenic or likely pathogenic alternate alleles were supported in the blood, while five pathogenic or likely pathogenic alternate alleles were supported in the tumour (**Figure 2**). While five of these alleles were previously linked to *PIK3CA*-related overgrowth spectrum and/or megalencephaly capillary malformation polymicrogyria (MCAP) syndrome (**Table 1**), the pathogenic alternate alleles here were likely to be sequencing errors, as they were supported only by single reads.

**Table 1:** Pathogenic alternate alleles detected in *PIK3CA* in the tumour using VarScan.

ClinVar variation ID	HGVSp	Classification	Associated conditions	Supporting reads/total reads (VAF)
280875	ENSP00000263967.3:p.Arg108His	Pathogenic/likely pathogenic	MCAP syndrome Cowden syndrome	1/41 (2.4%)
376049	ENSP00000263967.3:p.Arg88Gln	Pathogenic	MCAP syndrome Cowden syndrome PROS	1/43 (2.3%)
809567	ENSP00000263967.3:p.Gly364Arg	Pathogenic/likely pathogenic	MCAP syndrome PROS	1/51 (1.9%)
376243	ENSP00000263967.3:p.Pro539Arg	Likely pathogenic	MCAP syndrome	1/39 (2.5%)
3774497	ENSP00000263967.3:p.Gln546Leu	Likely pathogenic	PROS	1/36 (2.7%)

MCAP, megalencephaly capillary malformation polymicrogyria; PROS, *PIK3CA*-related overgrowth spectrum; VAF, variant allele frequency.

No somatic SNVs or indels that may activate *PIK3CA* were reliably detected. No somatic structural variants affecting the *PIK3CA* gene, or its promoter or associated enhancers, were detected in this analysis. Somatic copy number analysis reported *PIK3CA* as diploid. Overall, no somatic SNVs, indels, structural variants, or copy number alterations that are known to activate *PIK3CA* were detected.

### The mutational landscape of the tumour is consistent with that of previously reported urothelial carcinomas

**Figure 3:**
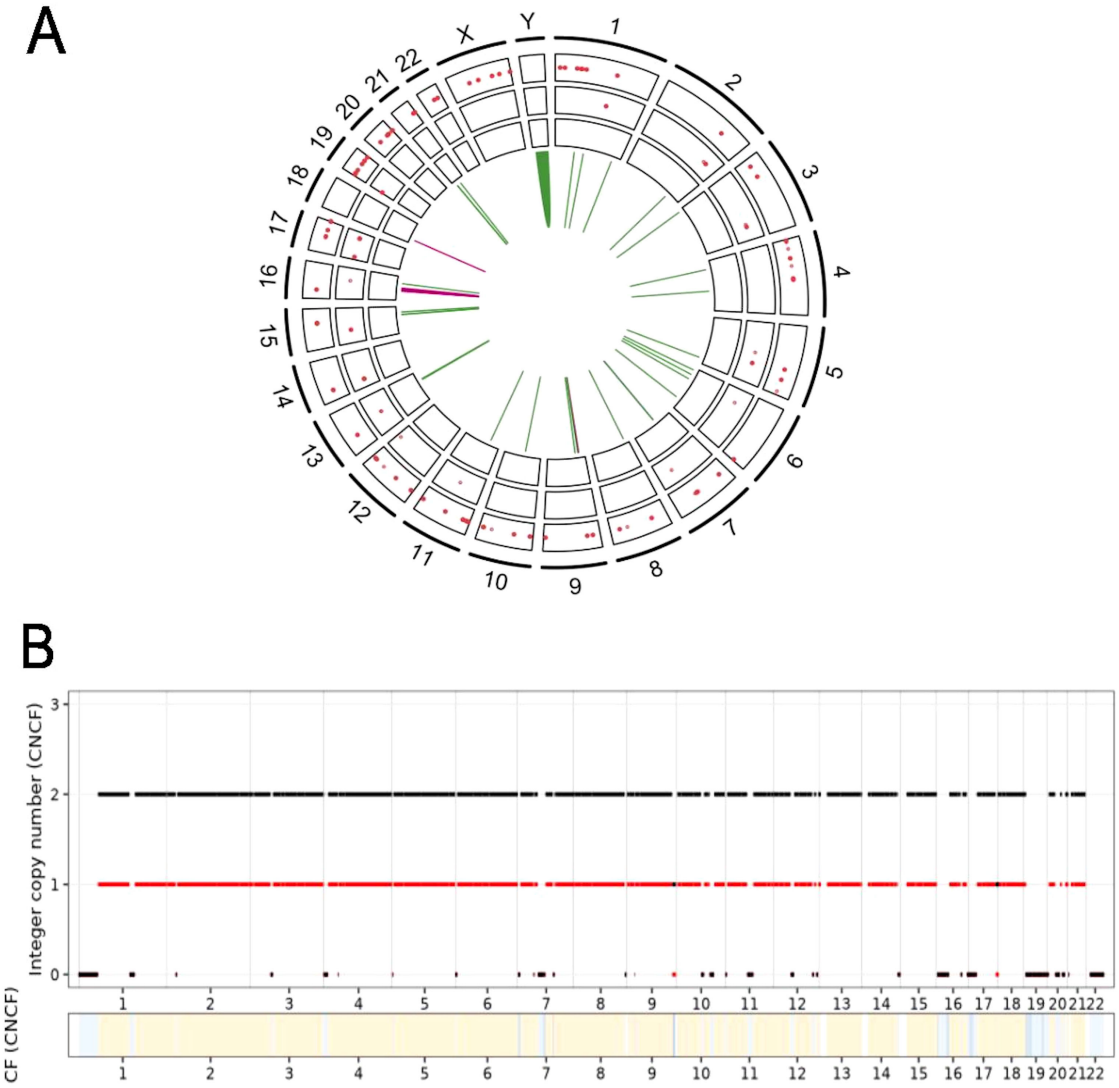
**(A)** Small non-synonymous somatic variants and structural variants detected genome-wide. SNVs are in the outer ring, indels in the middle ring, and MNVs in the inner ring. Structural variants are in the centre (green, inversions; pink, deletions; blue, duplications). **(B)** Somatic copy number alterations (black, total copy number; red, minor copy number; CF, cellular fraction). SNVs, single-nucleotide variants; indels, insertions and deletions; MNVs, multinucleotide variants.

Of the 12,691 somatic small mutations detected genome-wide, one driver mutation was identified. An SNV in *MAP2K1* resulting in a gain-of-function mutation in the MEK1 protein (P124L) was detected at a variant allele frequency of 17%. MEK1 is an activator of the MAPK/ERK pathway. Structural variant analysis detected 33 SVs (28 inversions, four deletions, and one duplication) (**Figure 3A**). Of all SVs, 12 affected one or more protein-coding genes (**Table 2**). A large 804-kb deletion including *CDKN2A* and *CDKN2B* was detected with an estimated variant allele frequency (VAF) of 12%. Somatic copy number alteration (sCNA) analysis confirmed a homozygous loss within chromosome 9p containing *CDKN2A* and *CDKN2B* present at a frequency of 21% in the tumour sample (**Figure 3B**). *CDKN2A* and *CDKN2B* are tumour suppressor genes, with *CDKN2A* deleted in up to 42% of urothelial carcinoma cases, with *CDKN2A/B* deletions co-occurring in 23% of cases (9, 10). Additional homozygous deletions within chromosomes 3p, 17p, 19p, 19q, and 22q were detected, resulting in losses in multiple genes that are also recurrently altered in urothelial carcinoma, including *ERCC2*, *CREBBP*, *KMT2D*, *PRMD4*, and *SETD2* (9, 10). Heterozygous loss of *TSC1* was also observed. No gains were detected (**Figure 3B**). *CREBBP*, *KMT2D*, and *SETD2* are chromatin remodelling genes, while *PRMD4* and *TSC1* are both tumour suppressors that regulate the PI3K pathway (11, 12). Overall, the small somatic driver mutation detected in *MAP2K1* in this tumour is a rarer occurrence in urothelial carcinoma cases, while the somatic copy number profile is consistent with previous findings in urothelial carcinoma.

**Table 2:** Structural variants (SVs) affecting protein-coding genes detected in the tumour.

SV type	Chromosome	Start position	End position	Size (bp)	VAF	Genes affected
INV	chr1	107889975	107890614	639	16%	*VAV3*
INV	chr5	161523567	161524516	949	19%	*GABRB2*
INV	chr6	28507581	28508810	1,229	20%	*GPX6*
INV	chr6	47964006	47964686	680	9%	*PTCHD4*
INV	chr6	132448638	132449203	565	12%	*STX7*
INV	chr8	14192656	14193735	1079	10%	*SGCZ*
DEL	chr9	21908255	22712570	804,315	12%	*MTAP*, *CDKN2A*, *CDKN2B*, *DMRTA1*
INV	chr9	36076171	36076902	731	24%	*RECK*
INV	chr10	60045041	60045975	934	17%	*ANK3*
INV	chr13	77927036	77927857	821	12%	*EDNRB*
INV	chr15	71034937	71035459	522	21%	*LRRC49*
INV	chrY	11326459	56843845	45,517,386	2%	*USP9Y*, *DDX3Y*, *UTY*, *TMSB4Y*, *VCY*, *VCY1B*, *NLGN4Y*, *CDY2B*, *CDY2A*, *HSFY1*, *HSFY2*, *KDM5D*, *EIF1AY*, *RPS4Y2*, *PRORY*, *RBMY1B*, *RBMY1A1*, *RBMY1D*, *RBMY1E*, *PRY2*, *RBMY1F*, *RBMY1J*, *PRY*, *BPY2 DAZ1*, *DAZ2*, *CDY1B*, *BPY2B*, *DAZ3*, *DAZ4*, *BPY2C*, *CDY1*

INV, inversion; DEL, deletion; VAF, variant allele frequency.

## Discussion

Mutations in the PI3K–AKT–mTOR pathway have been linked to benign overgrowth disorders, including hemimegalencephaly and syndromes belonging to the *PIK3CA*-related overgrowth spectrum (PROS), such as the Klippel–Trenaunay syndrome and CLOVES syndrome (13, 14). Somatic *PIK3CA* mutations frequently occur across many cancer types; however, evidence linking CLOVES syndrome to increased cancer risk is not conclusive. In a study of 122 patients, it was found that patients with CLOVES syndrome are at significantly higher risk of developing Wilms tumour, which is an embryonic renal cancer (incidence 3.3% in CLOVES vs. 0.0001% in the general population); however, the *PIK3CA* mutational status of these tumours was not reported (15). In another study of 267 PROS patients, six (2.2%) patients presented with cancer, including two paediatric and four adult cancers. Ultradeep sequencing was performed on 4/6 tumours; *PIK3CA* mutations were confirmed in one paediatric and one adult tumour (7). The relationship between CLOVES syndrome and cancer risk remains unclear. Here, whole-genome sequencing could not confirm the presence of a *PIK3CA* mutation in this patient’s primary urothelial carcinoma, nor were regulatory or structural alterations affecting this gene observed. Rather, a gain-of-function driver mutation in *MAP2K1* was detected, which would promote signalling through the MAPK/ERK pathway via hyperactivation of MEK1. Mutations in *MAP2K1* are rare across bladder cancers, occurring in 0.5% of bladder cancer samples in The Cancer Genome Atlas database. However, the regulation of the PI3K pathway is nonetheless disrupted in this tumour due to losses in *PRMD4* and *TSC1*. Losses of these genes are common in urothelial carcinoma, as are homozygous deletions of *CDKN2A* and *CDKN2B*, both of which were also detected in this tumour. As sCNAs recurrently altered in urothelial carcinoma were detected in this tumour, and no *PIK3CA* activating mutations were detected, this tumour may have arisen independently of CLOVES syndrome.

A limitation of this study was the availability of only a single-region, low-purity tumour sample for genomic analyses. Intra-tumoural heterogeneity could not be investigated in this study, as multi-region samples of the tumour were not available. Average WGS coverages of 33× and 34× were achieved for tumour and blood samples, respectively, which may limit the ability to detect *PIK3CA*-mutated subclones. Potentially, ultradeep sequencing could have uncovered low-frequency *PIK3CA* mutations in this patient’s blood or tumour. Moreover, contamination of the tumour sample with normal tissues may have limited the ability to detect low-frequency somatic mutations (16). However, as *PIK3CA* mutations are hypothesised to be a potential driver of this patient’s tumour, such a mutation was hypothesised to be present in the tumour at a frequency detectable by the WGS data generated here.

A potential explanation for an unchanged cancer risk in CLOVES syndrome patients is that the tissue distribution of *PIK3CA* mutations in CLOVES syndrome differs from that of cancers carrying *PIK3CA* mutations. Overgrowths in CLOVES syndrome predominantly occur in tissues of mesodermal and neuroectodermal origins, while *PIK3CA*-mutated cancers occur in tissues derived from ectodermal and endodermal tissues (3). Alternatively, it has been proposed that *PIK3CA* mutations alone are not adequate tumour-initiating events (3). There is evidence to suggest that this may be the case in urothelial carcinoma; in murine models of bladder cancer, increased urothelial thickness and nuclear atypia were observed in *PIK3CA*^H1047^ mice, but this did not progress to invasive disease, indicating that additional genetic alterations are necessary for tumourigenesis in this context (17). While we did not detect gain-of-function mutations in *PIK3CA* in this case, we did observe other alterations that are characteristic of urothelial carcinoma, such as *CDKN2A/B* deletions and heterozygous loss of *TSC1*. Further investigation is required to conclusively determine cancer risk in patients with CLOVES syndrome.

## Conclusion

In this case report, the whole-genome sequencing of a urothelial carcinoma diagnosed in a patient with CLOVES syndrome identified somatic variants consistent with those of previously reported urothelial carcinoma cases. No strong evidence of activating mutations affecting *PIK3CA* was detected in the tumour, which suggests that this patient’s urothelial carcinoma was not driven by somatic mosaic *PIK3CA* mutations related to CLOVES syndrome. However, multi-region sampling and/or ultradeep sequencing would be required to ensure that *PIK3CA*-mutated subclones do not remain undetected and that low-frequency *PIK3CA* variants are thoroughly investigated.

## Data Availability

The datasets presented in this study can be found online at https://figshare.com via thefollowing links:https://doi.org/10.6084/m9.figshare.30096529.v2https://doi.org/10.6084/m9.figshare.30102946.v1https://doi.org/10.6084/m9.figshare.30096523.v1https://doi.org/10.6084/m9.figshare.30096415.v1.

## Supplementary material

Supplementary Figure 1 Integrative Genomics Viewer screenshots of *PIK3CA* hotspot mutations. No strong alternatie allele support at hotspot locations **(A)** E452 and E545 in exon 9, **(B)** H1047 in exon 20 and **(C)** N345 in exon 5.
