# Supplementary material for: Case Report: Whole-genome sequencing of urothelial carcinoma in an adult patient with CLOVES syndrome reveals a lack of PIK3CA mutation and a genomic landscape consistent with urothelial carcinoma
Source: Front Oncol. 2026 Feb 20;16:1704090. doi: 10.3389/fonc.2026.1704090 (PMC12962928; doi:10.3389/fonc.2026.1704090)
Supplement: Supplementary Figure 1 — Integrative Genomics Viewer screenshots of PIK3CA hotspot mutations. No strong alternatie allele support at hotspot locations (A) E452 and E545 in exon 9, (B) H1047 in exon 20 and (C) N345 in exon 5. [file DataSheet1.pdf]

Supplementary Materials and Methods

Sample acquisition, sample processing, and whole genome sequencing

Tissue for whole genome sequencing was available from the primary tumour resection. Matched blood was also acquired for tumour-normal comparisons. DNA was extracted from tumour using the AllPrep DNA minikit (Qiagen, Hilden, Germany) and matched blood samples using a DNA blood minikit (Qiagen). DNA was quantified by Quibit fluorometer (Invitrogen, Carlsbad, CA, USA). DNA integrity was assessed by agarose gel electrophoresis. Paired end (100bp) whole genome sequencing reads were generated by sequencing on the BGI Genomics DNA Nanoball sequencing platform.

Paired end read filtering and alignment

SOAPnuke was used to filter adaptor sequence, contamination, and low-quality base pair reads from lane level raw sequencing reads using parameters "-n 0.001 | 10 -adaMR 0.25" (1). Lane level reads were aligned to the human reference genome (GRCh38) using the Burrows-Wheeler Aligner mem (v0.7.17). Duplicate reads were marked using GATK4 (v4.2.0) MarkDuplicates. For each sample, aligned lane level reads were sorted using samtools (v1.9) and merged using MergeSamFiles. Quality scores of sample level BAM files were recalibrated using BaseQualityScoreRecalibration with the following reference files provided to parameter "—known-sites''; Homo_sapiens_assembly38.dbsnp138.vcf.gz, Homo_sapiens_assembly38.known_indels.vcf.gz and Mills_and_1000G_gold_standard.indels.hg38.vcf.gz.

Somatic mutation calling

SNVs and indels were detected using Mutect2 (v.4.2.0) and Strelka (v.2.9.10) from the matched normal and tumour pair (2,3). Mutect2 was run using a panel of normals provided by the Broad Institute and the gnomAD germline population reference to remove common variants and mapping artifacts. To filter potential false positive calls from Mutect2, GATK LearnReadOrientationModel was applied to remove mutations with FFPE strand bias. GetPileupSummaries and CalculateContamination were ran to calculate cross contamination. Known germline common variants from ExAC were provided to GetPileupSummaries. FilterMutectCalls was ran with default parameters. Mutect was ran in forcecalling mode for hotspot mutations by providing a VCF *PIK3CA* hotspot mutations to the --alleles option. To improve Strelka's performance, Manta (v.1.6.0) was initially ran using default settings to detect indels and structural variants (4). BCFtools (v.1.12) was used to left align and normalise indels detected by Manta. These indels were

subsequently used as input to Strelka using –indelCandidates. All somatic mutation calling was restricted to chromosomes 1-22, X, Y, M. BCFtools norm was used to left align and normalise indels from Mutect2 and Strelka separately and then PASS variants were selected. SnpSift annotate was used to remove common variants in the dbSNP database (version 138). Filtered variant call sets were combined into one VCF file using GATK3 (v3.8.1) CombineVariants -genotypeMergeOptions set to PRIORITIZE and the --rod_priority_list set to mutect2, strelka. Multiallelic sites were split using vt decompose. Variant annotation was performed using variant effect predictor (v96) and filter_vep was used to select mutations affecting canonical protein coding transcripts (--filter "Biotype is protein_coding" and --filter "CANONICAL is YES").

To ensure low-frequency variants in *PIK3CA* were not missed by strict filters, VarScan (v2.3.9) was used to count the number of alternate alleles detected at each position across the gene in both tumour and blood samples separately (5). Samtools mpileup was used to generate pileup files (--no-BAQ -d 0 -q 10 -Q 15) for the tumour and matched blood samples separately. These pileups were used as input to VarScan (–min-coverage-normal 10 –min-coverage-tumour 10 –min-var-freq 0.005). The –validation flag was set to output all positions even if not expected to be variant. All alternate alleles detected across *PIK3CA* were annotated with pathogenicity classifications using VEP.

Identification of driver mutations

Cancer Genome Interpreter was used to annotate SNVs, indels, and MNVs as passenger or driver mutations. Only driver mutations also reported by OncoKB and/or ClinVar were deemed as driver mutations.

Somatic copy number calling

Pileup files were generated for tumour and matched normal BAM files using snp-pileup with parameters "-q15 -Q20 -P100 -r25,0". Pileup files were used as input to FACETS Suite (v.2.0.9) and FACETs (v.0.6.1) to perform allele-specific copy number calling (--purity-cval 1500 and –cval 750) (6).

Somatic structural variant calling

DELLY2 was used to detect structural variants from the tumour-normal pair using a minimum mapping quality of 20 (7). Outputs of DELLY were filtered for SVs with a minimum 20X coverage in tumour, minimum length of 500bp, and minimum allele frequency of 0.01. PASS variants were selected. AnnotSV was used to annotate SVs (v3.5.2).

Germline variant calling

GATK HaplotypeCaller (v4.3.0.0) was used to detect SNVs and indels across a panel of 49 genes in which germline variants are associated with increased UC risk (8) and in the *PIK3CA* gene. The genomic coordinates of these 50 genes and of enhancers and promoters associated with them were provided to HaplotypeCaller in a BED file using the –intervals flag. Variants were annotated with pathogenicity classifications using VEP. DELLY (v1.1.6) was used to detect germline structural variants and copy number variants. Structural variants were annotated with AnnotSV (v3.5.2). Germline copy number variants shared between tumour and normal tissues were overlapped with the gene panel previously described using GRanges in R.

References

1. Chen Y, Chen Y, Shi C, Huang Z, Zhang Y, Li S, Li Y, Ye J, Yu C, Li Z, et al. SOAPnuke: a MapReduce acceleration-supported software for integrated quality control and preprocessing of high-throughput sequencing data. *Gigascience* (2017) 7:gix120. doi: 10.1093/GIGASCIENCE/GIX120

2. Benjamin D, Sato T, Cibulskis K, Getz G, Stewart C, Lichtenstein L. Calling Somatic SNVs and Indels with Mutect2. *bioRxiv* (2019)861054. doi: 10.1101/861054

3. Saunders CT, Wong WSW, Swamy S, Becq J, Murray LJ, Cheetham RK. Strelka: accurate somatic small-variant calling from sequenced tumor–normal sample pairs. *Bioinformatics* (2012) 28:1811–1817. doi: 10.1093/BIOINFORMATICS/BTS271

4. Chen X, Schulz-Trieglaff O, Shaw R, Barnes B, Schlesinger F, Källberg M, Cox AJ, Kruglyak S, Saunders CT. Manta: Rapid detection of structural variants and indels for germline and cancer sequencing applications. *Bioinformatics* (2016) 32:1220–1222. doi: 10.1093/BIOINFORMATICS/BTV710,

5. Koboldt DC, Chen K, Wylie T, Larson DE, McLellan MD, Mardis ER, Weinstock GM, Wilson RK, Ding L. VarScan: variant detection in massively parallel sequencing of individual and pooled samples. *Bioinformatics* (2009) 25:2283. doi: 10.1093/BIOINFORMATICS/BTP373

6. Shen R, Seshan VE. FACETS: allele-specific copy number and clonal heterogeneity analysis tool for high-throughput DNA sequencing. *Nucleic Acids Res* (2016) 44:e131. doi: 10.1093/NAR/GKW520

7. Rausch T, Zichner T, Schlattl A, Stütz AM, Benes V, Korbel JO. DELLY: structural variant discovery by integrated paired-end and split-read analysis. *Bioinformatics* (2012) 28:i333–i339. doi: 10.1093/BIOINFORMATICS/BTS378

8. Nassar AH, Abou Alaiwi S, AlDubayan SH, Moore N, Mouw KW, Kwiatkowski DJ, Choueiri TK, Curran C, Berchuck JE, Harshman LC, et al. Prevalence of pathogenic germline cancer risk variants in high-risk urothelial carcinoma. *Genetics in Medicine* (2020) 22:709–718. doi: 10.1038/s41436-019-0720-x
