# Supplementary figures and images for: Case Report: Whole-genome sequencing of urothelial carcinoma in an adult patient with CLOVES syndrome reveals a lack of PIK3CA mutation and a genomic landscape consistent with urothelial carcinoma
Source: Front Oncol. 2026 Feb 20;16:1704090. doi: 10.3389/fonc.2026.1704090 (PMC12962928; doi:10.3389/fonc.2026.1704090)

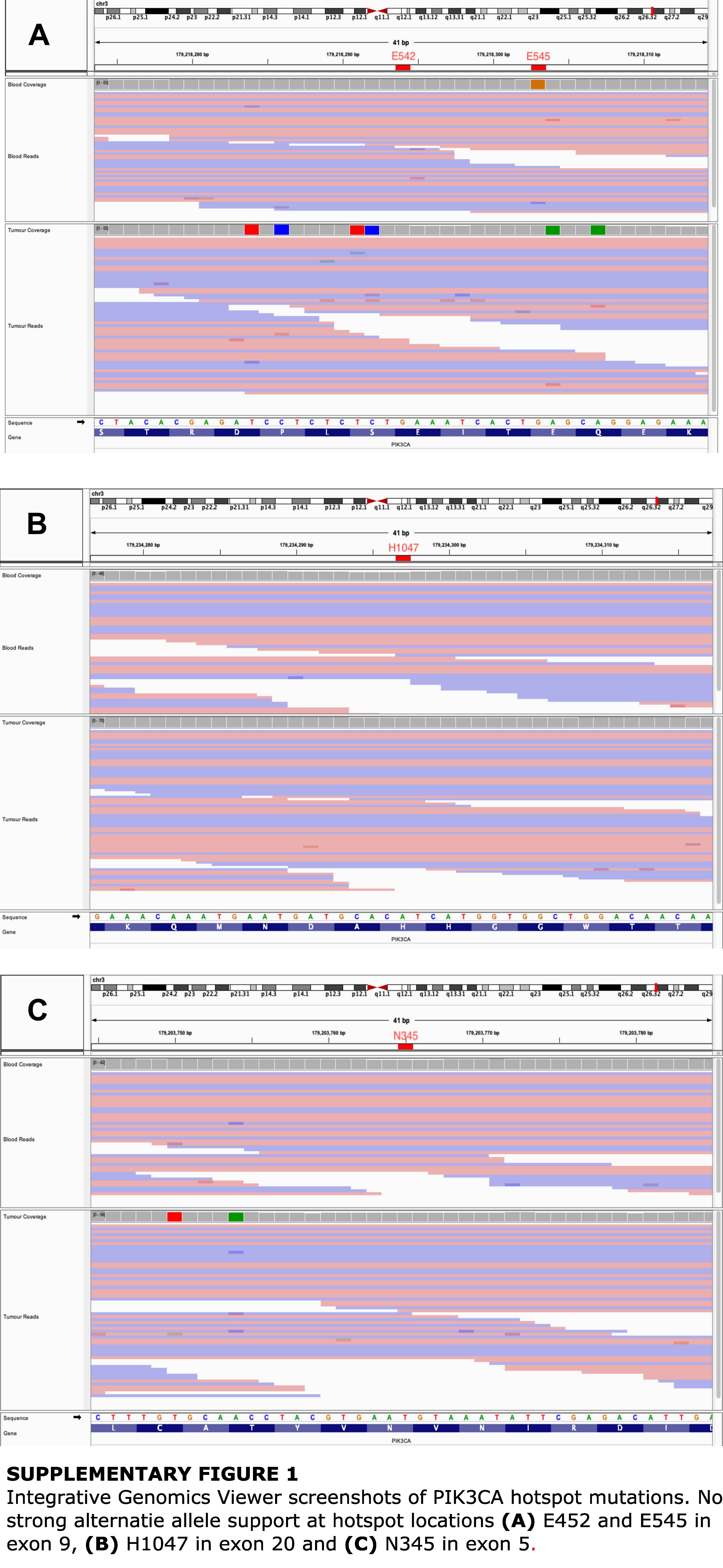

Supplement: Supplementary file 2 [file Image1.jpg]
